# Human Tonic and Phasic Smooth Muscle Myosin Isoforms Are Unresponsive to the Loop 1 Insert

**DOI:** 10.1155/2013/634341

**Published:** 2013-11-05

**Authors:** Katalin Ajtai, Azad Mayanglambam, Yihua Wang, Thomas P. Burghardt

**Affiliations:** 1 Department of Biochemistry and Molecular Biology, Mayo Clinic Rochester, 200 First Street SW, Rochester, MN 55905, USA; 2 Department of Physiology and Biomedical Engineering, Mayo Clinic Rochester, 200 First Street SW, Rochester, MN 55905, USA

## Abstract

Smooth muscle myosin gene products include two isoforms, SMA and SMB, differing by a 7-residue peptide in loop 1 (i7) at the myosin active site where ATP is hydrolyzed. Using chicken isoforms, previous work indicated that the i7 deletion in SMA prolongs strong actin binding by inhibiting active site ingress and egress of nucleotide when compared to i7 inserted SMB. Additionally, i7 deletion inhibits Pi release associated with the switch 2 closed → open transition in actin-activated ATPase. Switch 2 is far from loop 1 indicating i7 deletion has an allosteric effect on Pi release. Chicken SMA and SMB have unknown and robust nucleotide-sensitive tryptophan (NST) fluorescence increments, respectively. Human SMA and SMB both lack NST increments while Pi release in Ca^2+^ ATPase is not impacted by i7 deletion. The NST reports relay helix movement following conformation change in switch 2 but in the open → closed transition. The NST is common to all known myosin isoforms except human smooth muscle. Other independent works on human SMA and SMB motility indicate no functional effect of i7 deletion. Smooth muscle myosin is a stunning example of species-specific myosin structure/function divergence underscoring the danger in extrapolating disease-linked mutant effects on myosin across species.

## 1. Introduction

Myosin is the chemomechanical energy transducer in muscle hydrolyzing ATP to power actin movement during contraction. It has a globular head domain called subfragment 1 (S1) and a tail domain that forms dimers. S1 contains an active site for ATP hydrolysis, an actin binding site, and a lever-arm whose rotary movement cyclically applies tension to cause contractility while myosin is strongly actin bound. Myosin energy transduction starts in the ATP binding site where peptides are linked to the 7-stranded *β*-sheet sense position and coordination of the ATP *γ*-phosphate [1, 2]. ATP hydrolysis occurs rapidly, but products are not released until actin binds. The C-loop portion of the actin binding site senses actin contact that is transmitted to two strands in the 7-stranded *β*-sheet, releasing product with the opening of switch 2 and translating the relay *α*-helix [3]. Relay helix linear movement, sensed by the nucleotide sensitive tryptophan (NST) [4, 5], impinges on the converter changing linear force into torque to rotate the lever-arm. The lever-arm converts torque generated in the motor into the linear step-size displacement [6] and undergoes shear strain due to the resisting load [7]. Strain is shared among the lever-arm and the bound myosin light chains, essential (ELC) and regulatory (RLC). In smooth muscle myosin, phosphorylation of RLC regulates actin activation of myosin ATPase. Heavy meromyosin (HMM) contains the first ~1300 residues of the myosin heavy chain(MHC). It binds ELC and RLC and forms dimers. We use here an engineered short human smooth muscle (aorta) S1 containing the first 813 residues of the MHC. Short S1 has ELC but not RLC binding sites and the molecule is a monomer containing MHC and ELC. Without RLC, short S1 is always “on” for actin activation [8].

Smooth muscle myosin isoform variants have distinct heavy chain (MHC) sequences (gene MYH11) associating with one of two distinct ELCs. MHC sequences vary at the C-terminus with terminal peptides differing in sequence and in length [9]. MHC sequences also vary in S1 at the active site in loop 1. A7-residue peptide from loop 1 (i7) in the B-isoform (SMB) is deleted in the A-isoform (SMA). SMA and SMB isoforms were compared functionally in chicken HMM constructs. The *V*_max_ in actin-activated ATPase and the *in vitro* motility velocity were inhibited by the i7 deletion in SMA indicating inhibition of rate-limiting phosphate (Pi) release and ADP dissociation [10]. These effects were independent of the ELC isoform bound to HMM. Mechanical properties of single HMMs, including unitary step size and force, are indistinguishable while kinetics related to the ingress and egress of nucleotide to the active site are inhibited by the i7 deletion [11].

Under saturating ATP conditions, inhibited ADP release by the i7 deletion prolongs strong actin binding time, *t*_on_, slowing the *in vitro* motility. It is unknown how shortened loop 1 inhibits Pi release because Pi probably leaves the active site by a pathway alternative to reversing its ingress [12, 13]. The i7 deletion inhibition of Pi release suggests influencing regions of myosin beyond the active site and near switch 2 where it can also affect the relay helix and the NST. We proposed that the i7 deletion in SMA influences the energy transduction machinery in the relay helix because our human short S1 SMA construct, sSMA, lacked NST sensitivity to ATP hydrolysis [8]. sSMA is the only myosin isoform known to lack NST sensitivity to ATP hydrolysis. Our objective here is to test if the effect of i7 deletion penetrates beyond inhibiting active site access by perturbing a distant myosin transduction mechanism.

We investigated the significance of loop 1 length in NST sensitivity to ATP hydrolysis with new human short S1 constructs containing i7 (sSMB). A new protein purification protocol was developed to better remove contaminants potentially perturbing quantitative fluorescence increments and applied to all constructs. We found that WT sSMA and sSMB proteins both have a significant fluorescence increment of ~18% with ATP binding to the active site as reported previously for sSMA and where we showed that the increment is not due to the NST at W506 [8]. Mutant sSMA-W506 and the new mutant construct sSMB-W506 have 6 of 7 tryptophans replaced with phenylalanine or methionine. The remaining tryptophan is the NST at W506. The mutants have identical and minimal increments of ~11% compared to the ~36% increment for the chicken SMB construct analogous to sSMB-W506 [14]. The findings imply that the human MYH11 gene product is unique because its W506 is not sensitive to nucleotide binding and hydrolysis while chicken SMB and several other myosin II’s have the NST. In human SMA, the i7 deletion does not affect the wider myosin energy transduction mechanism. Considering available data on human SMA and SMB, we conclude that the functional effect of the i7 deletion in human SMA has not yet been identified.

## 2. Materials and Methods

### 2.1. Chemicals

Ammonium sulfate (BioXtra), ATP, ADP, *β*-mercaptoethanol (BME), Na-Azide, dithiothreitol (DTT), and phenylmethylsulfonyl fluoride (PMSF) were from Sigma (St. Louis, MO, USA). Phalloidin was from Life Technologies (Grand Island, NY, USA). Bradford protein concentration assay was from Bio-Rad (Hercules, CA, USA). Leupeptin, chymostatin, and pepstatin were from Peptides International (Louisville, KY, USA). All chemicals were of reagent grade or ultrapure if available. The His-tag mouse monoclonal and anti-mouse goat IgA antibodies for the motility assay were from Abgent (San Diego, CA, USA) and Southern Biotech (Birmingham, AL, USA), respectively.

### 2.2. Cloning of Human Smooth Muscle Myosin Heavy Chain (MYH11) and Essential Light Chain (MYL6)

The cloning of MHC and ELC in sSMA was carried out as described in [8].

### 2.3. Site-Directed Mutagenesis

We sequentially mutated six of the seven endogenous tryptophans in sSMA to phenylalanine or methionine using QuikChange Mutagenesis (Stratagene, La Jolla, CA, USA) to produce a mutant heavy chain containing a single tryptophan residue at position 506 as described in [8]. The mutant, sSMA-W506, had W33, W37, W435, W591, and W619 being changed to phenylalanine while W540 was changed to methionine.

The 7-amino acid extension of the loop 1 region in the sSMA and sSMA-W506 heavy chains (QGPSFSF between T211 and G212) was inserted by site-directed mutagenesis using a “two-step” QuickChange method (Stratagene, La Jolla, CA, USA). The mutant DNA was subcloned into vector pV1392 (Invitrogen). The QGPSFSF insertions into loop 1 of sSMA and sSMA-W506 produced sSMB and sSMB-W506.

Baculoviruses were prepared for SF9 cell infection using the Bac-to-Bac system. Virus and protein productions were done at the Baculovirus/Monoclonal Antibody Core Facility, Baylor College of Medicine, Houston, TX, USA.

Construct accuracies were verified by DNA sequencing and mass spectrometry.

### 2.4. Protein Preparation

Protein preparation and purification were done as described in [8] but with one substantial change. The Ni-NTA agarose purification step following acto-S1 sedimentation was replaced with MonoQ FPLC (MonoQ5/50GL GE Healthcare) chromatography.

Pelleted acto-S1 was resuspended in 5mL of 20mM Tris pH 7.5, 0.2 M KCl, 2mM MgCl_2_, 2mM BME, 0.5mM PMSF, 0.01 mg/mL leupeptin, and 1mM ATP and dialyzed overnight in this buffer. The following morning, 1mM ATP was added to the sample to dissociate actin that was then centrifuged at 330600 ×g for 65 min. 5 mM pH 8.0 EDTA was added to the supernatant that was then incubated for 5 min on ice then applied to a PD-10 column equilibrated in Buffer A consisting of 30 mM Tris-HCl pH 7.4, 2mM BME, and 1 *μ*g/mL leupeptin. 5mM pH 8.0 EDTA was added to the crude protein sample and incubated on ice for 1 hour. The EDTA and PD-10 treatments remove ATP or ADP and change buffer. A linear gradient elution between 0 and 0.35 M KCl in Buffer A was used for the FPLC. We routinely loaded 6–7 mg protein on the MonoQ column for purification yielding 0.5–1 mg of purified protein.

The ~0.5 mg protein eluted from the column was dialyzed overnight into Measuring Buffer (see below) and then used in spectroscopic experiments or precipitated in a dialysis solution of 25 mM TES pH 7.0, 1mM EDTA, 1mM DTT, 1 *μ*g/mL leupeptin, and 75% ammonium sulfate. The precipitate was collected via centrifugation and stored at −20°C for 2–3 months with no loss of specific ATPase activity.

### 2.5. ATPase Assays

K^+^EDTA-ATPase and Ca^2+^-ATPase assays were performed as described in [8].

### 2.6. Spectroscopy Measurements

Measuring buffer consisted of 0.45 M KCl, 20mM Tris-HCl pH 7.5, 2mM MgCl_2_, and 0.5 mM DTT. ADP or ATP bound states were formed by incubating the proteins at concentrations of 0.8–8.0 *μ*M in measuring buffer with 0.133 mM ADP or 0.1 mM ATP for 3 minutes at 20°C. Apo protein was treated identically but without addition of nucleotide.

Steady-state fluorescence measurement results use the fluorescence increment, Δ*F*, defined by

(1) $$\Delta F=\frac{F\;(\text{nucleotide})-F(\text{Apo})}{F\;(\text{Apo})},$$

where *F* is fluorescence intensity in the presence (nucleotide) or absence (Apo) of ATP or ADP. Fluorescence intensity measurements were performed as described in [8].

### 2.7. In Vitro Motility Measurements

The gliding velocity of rhodamine-labeled phalloidin-F-actin (Rh-phalloidin-F-actin) over surface bound sSMA or sSMB constructs (*in vitro* motility assay) was performed at 22°C as in [3] with a minor modification. 10 mM MOPS replaced imidazole buffer to avoid competition between buffer and the C-terminal His tag for the His antibody (from mouse) attached to the nitrocellulose layer in the flow cell. Both sSMA and sSMB bound the Rh-phalloidin-F-actin normally but failed to make it move. Knowing that our constructs have a short lever-arm and that the distance and the mode of the attachment of the constructs to the surface affect motility [15], we elongated the span between the C-terminal His tag and the nitrocellulose surface of the coverslip by adding a second antibody (anti-mouse specificity from goat). This assay started first with the attachment of a mouse-specific antibody (made in goats) to the coverslip followed by the His-specific mouse antibody binding His-tagged sSMA or sSMB. We presumed that the double antibody linker gave more freedom to the myosin heads to interact with the rhodamine labeled F-actin. Again, actin binding but no motility was observed at 25 or 60 mM KCl and at different S1 surface densities produced from S1 bulk concentrations of 0.8–5.0 *μ*M. We concluded that the construct lever-arm was too short to move the actin filaments.

## 3. Results

### 3.1. Myosin Purification

**Figure 1:**
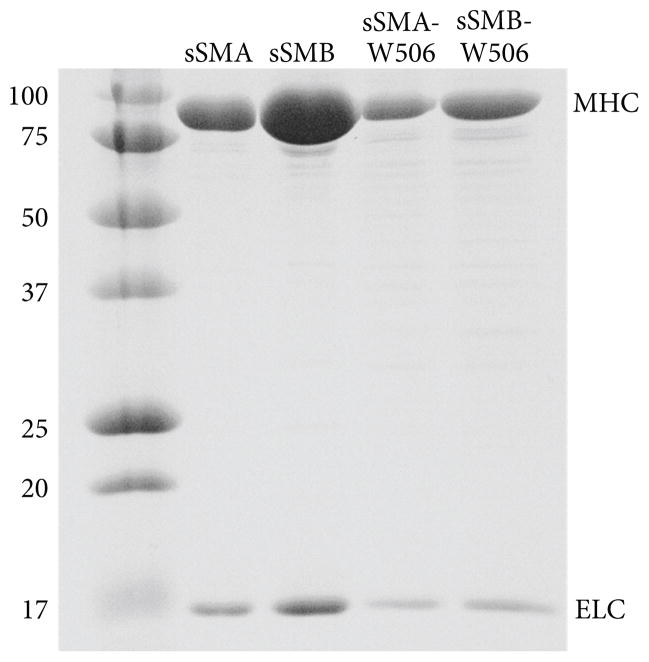
Coomassie-stained SDS-PAGE for each of the expressed proteins used in this study. Mass listed on the left is in kD. The MHC and ELC protein bands dominate the transmitted light image for each isoform confirming purity.

Figure 1 compares Coomassie-stained SDS-PAGE for all the expressed proteins used in this study. The MHC and ELC protein bands dominate the transmitted light image for each isoform confirming purity. The purification protocol using FPLC described in Section 2.4 provided significant improvement in purity of the mutants, sSMA-W506 and sSMB-W506, compared to the protocol using the C-terminal His-tag described previously in [8]. Purity of the native isoforms, sSMA and sSMB, was not significantly improved by the new protocol. The relative purities between FPLC and His-tag purification protocols are also reflected in the slightly higher NST increment in the sSMA-W506 isoform compared to previous results. A larger fraction of protein contaminants with the His-tag purification for sSMA-W506 probably lowered the fluorescence increment (1) by increasing the denominator while leaving the numerator unchanged because they contained tryptophan that did not respond to nucleotide.

### 3.2. Myosin ATPases

Table 1 compares the K^+^EDTA and Ca^2+^ ATPases for all the expressed proteins. ATPase is unaffected by the i7 insertion into SMA showing that it does not substantially affect the rate limiting step in these ATPases. ATP hydrolysis has been suggested to be rate limiting in skeletal myosin K^+^EDTA ATPase [16]. The hydrolysis rate is not impacted by nucleotide ingress and egress or Pi release; hence, the lack of K^+^EDTA ATPase sensitivity to i7 insertion is expected. Ca^2+^ ATPase in skeletal myosin has a phosphate burst [16] and actin activation [17] indicating that Pi release rate is probably rate limiting. The Ca^2+^ ATPase rate is expected to be lower in human SMA based on previous results with chicken SMA and SMB [10]. The Ca^2+^ ATPase implies that the i7 deletion is a local perturbant potentially affecting nucleotide access to the active site but not the wider myosin energy transduction mechanism.

### 3.3. Myosin Fluorescence Increments

Table 1 compares fluorescence increments (1) for all the expressed proteins. The sSMA and sSMB isoforms have 7 tryptophan residues and an equally robust increment in the presence of ATP that is comparable to skeletal S1 [18]. This indicates a strong cumulative sensitivity to nucleotide binding that if concentrated into the NST corresponds to a large fluorescence change in the residue. The mutant isoforms with a single tryptophan have a reduced increment that is likewise independent of i7. This indicates that W506 is not the NST in the human MYH11 (SMA or SMB). In comparison, WT chicken sSMB has an increment comparable to human SMA or SMB while the increment in its single tryptophan mutant analogous to our human sSMB-W506 is twofold larger [14]. The i7 insert in human MYH11 does not affect W506 nucleotide sensitivity appreciably suggesting that it does not impact the energy transduction pathway. These results are consistent with the Ca^2+^ ATPase results. Our data is consistent with the view that i7 deletion potentially hinders nucleotide access to the active site but does not influence the energy transduction pathways involving the relay helix.

### 3.4. Myosin In Vitro Motility

We attempted *in vitro* motility measurements on sSMA and sSMB using an anti-His antibody to bind the His-tagged C-term of the short S1 to the nitrocellulose coated surface. This system produced myosin-bound actin filaments but no motility. Next, we inserted a secondary antibody between the surface and the anti-His antibody to increase the distance from the surface to the myosin motor domain. This strategy produced actin movement with a longer S1 construct containing the two myosin light chains ELC and RLC [15] but again no motility with sSMA or sSMB. They have only ELC. We concluded that our short S1 constructs were unable to move actin in our assay because the lever-arm was too short.

## 4. Discussion

Functional consequences of the natural MYH11 product variations were investigated with focus on the effect of the loop 1 variant. Our objective was to test if the effect of the i7 deletion in sSMA penetrates beyond the active site into the imbedded and distant myosin transduction mechanism. In our experiments, we monitored Ca^2+^ ATPase rate and the NST fluorescence increment. The Ca^2+^ ATPase rate depends on the switch 2 closed → open transition that allows the Pi product to leave. Leaving product starts the lever-arm swing responsible for the power stroke. The NST increment detects the relay helix movement following conformation change in switch 2 but in the open → closed transition. The NST fluorescence increment follows ATP hydrolysis intermediates accompanying the lever-arm swing associated with cross-bridge repriming. Switch 2 and relay helix are far from the active site and loop 1 indicating that if i7 affects NST increment or Ca^2+^ ATPase rate, the effect is allosteric.

Previous work on chicken and rabbit MYH11 variants showed that insertion of i7 between T211 and G212 in SMA loop 1 upregulated *V*_max_ in actin-activated ATPase by ~2-fold and that deletion of the same peptide from SMB loop 1 downregulated *V*_max_ by ~2-fold. WT SMA and SMB proteins were from different organisms and probably had sequence differences other than i7, but quantitative comparisons were always between identical isoforms with and without i7. Actin activation involves a lowering of the activation energy barrier for the MgADP·Pi·M^**^ → MgADP·M^*^ + Pi transition for M^**^ and M^*^ myosin ATPase intermediates with different conformations corresponding to different NST fluorescence increments [19]. The actin binding and Pi release sites are distinct and distant from the active site implying that the change in *V*_max_ triggered by the SMB ↔ SMA interconversion is an allosteric activation ↔ inhibition.

Tissue-purified rat MYH11 gene products have motility velocity ~3-fold larger for SMB compared to SMA[20]. Other work on chicken MYH11 showed that step sizes for SMA and SMB were identical but that the time myosin is strongly actin-bound during its cycle, *t*_on_, differing by more than 2-fold due to i7 such that SMA has a larger *t*_on_. Given that the motility velocity *V*_motility_ = *d/t*_on_, the SMA has the lower motility velocity. Further investigation attributed the difference in *t*_on_ to active site accessibility [11]. The previous results indicated that the i7 affected nucleotide accessibility to the active site probably due to a steric inhibition by the shorter loop 1.

We studied the human MHY11 isoforms where SMA and SMB have identical sequences except at the site for the i7. The Ca^2+^ ATPase and the fluorescence increment do not signal a difference between SMA and SMB. We expected that the Ca^2+^ ATPase would be affected by the loop 1 insert because its rate-limiting step, Pi release, is affected by the i7 insert in actin-activated ATPase in the chicken SMA and SMB. We expected that the NST fluorescence increment would be affected by the i7 insert because it is absent in human sSMA [8] but present in chicken SMB [14]. Our findings indicate that either the human MYH11 is a unique MHC sequence in which the i7 deletion in SMA is at most a local perturbant possibly inhibiting access to the active site but not affecting the broader energy transduction mechanism or the impact of i7 is detectable only when myosin is in complex with actin.

Table 1 summarizes our findings and related data from the literature regarding ATPases (K^+^EDTA, Ca^2+^, and actin-activated Mg^2+^) and motility of the human SMA and SMB isoforms. We showed that our sSMA and sSMB constructs did not support actin motility due to their truncated lever arms. However, under identical experimental conditions, *V*_max_ and motility velocity are statistically identical for HMM constructs of human SMA and SMB [21, 22]. The loop 1 insert sequence for the human isoforms is variable and slightly different from our substituted sequence QGPSFSF, that is compared with EGPSFWR from which the *V*_max_ and motility velocity are derived [21] or QGPSFAY (accession NP 001035203). Considering all data in Table 1, it is likely that an i7 deletion effect in human SMA has not been identified. Various tests in the presence and absence of actin regarding the ingress and egress of nucleotide to the active site, ATP hydrolysis, Pi release, and tryptophan sensitivity to nucleotide binding and hydrolysis show no detectable effect of i7 on human smooth muscle myosin.

Our previous work on the human sSMA showed the lack of the NST fluorescence increment [8]. The NST is specifically named because its increment was observed and carefully characterized in many myosin isoforms. The human sSMA isoform was apparently different because W506 was not the NST and we suggested that the likely culprit was the deleted i7. We now know that both the human variants, sSMA and sSMB, do not have a normal NST although both proteins exhibit a normal fluorescence increment in the presence of ADP or ATP (Table 1).

## 5. Conclusions

MYH11 gene products include two isoforms differing by i7 at the active site. Using chicken and rabbit isoforms, laser-trap and *in vitro* motility studies indicate that i7 deletion in SMA prolongs strong actin binding by inhibiting active site ingress and egress of nucleotide when compared to i7 inserted SMB. This effect is probably due to the steric inhibition of nucleotide access to the active site. Actin-activated ATPase indicates that the i7 deletion also inhibits Pi release. Pi release in myosin is associated with the switch 2 closed → open transition. Switch 2 is far from the active site and loop 1 indicating that the i7 effect on Pi release is allosteric. Chicken SMA and SMB have unknown and robust nucleotide-sensitive tryptophan (NST) fluorescence increments, respectively. Using the human smooth muscle myosin, Pi release in Ca^2+^ ATPase and NST sensitivity to ATP hydrolysis were not impacted by i7 insertion into SMA. NST sensitivity to ATP hydrolysis is due to relay helix movement following conformation change in switch 2 but in the open → closed transition. The findings, together with other independent work on the human smooth muscle myosin isoforms found in the literature, indicate that a functional effect of i7 in human SMA has not been identified. Human smooth muscle myosin provides a stunning example of species-specific myosin structure/function divergence underscoring the ambiguity in extrapolating disease-linked mutant effects on myosin function across species.

## Figures and Tables

**Table 1:** MYH11 gene products SMA and SMB characterization in absence and presence of actin^a^.

	Parameter	Human		Chicken	
		SMA	SMB	SMA	SMB
1	Δ*F* (WT + ATP) %	18.44 ± 1.7	15.8 ± 1.9	u	18.4 [14]
2	Δ*F* (WT + ADP) %	5.7 ± 1.1	5.0 ± 1.0	u	12.1 [14]
3	Δ*F* (W506 + ATP) %	10.57 ± 0.6	11.65 ± 0.6	u	36.1 ± 1.1 [14]
4	Δ*F* (W506 + ADP) %	3.67 ± 1.7	6.97 ± 1.1	u	30.1 ± 2.1 [14]
5	Ca^2+^ ATPase WT (s^−1^)	3.62 ± 0.1	2.86 ± 0.33	u	5.9 ± 0.3 [3]
6	Ca^2+^ ATPase W506 (s^−1^)	1.02 ± 0.25	1.21 ± 0.15	u	u
7	EDTA ATPase WT(s^−1^)	4.45 ± 0.5	5.24 ± 0.11	u	14.0 ± 0.9 [3]
8	EDTA ATPase W506 (s^−1^)	3.52 ± 0.11	3.92 ± 0.52	u	u
9	*V*_max_ (s^−1^)	0.87 ± 0.07^b^ [22]	0.87 ± 0.09^b^ [21]	1.35 ± 0.39^c^ [10]	3.0 ± 0.34^c^ [10]
10	Motility (*μ*m/s)	0.38 ± 0.06^d^ [22]	0.36 ± 0.09^d^ [21]	0.49 ± 0.03 [11]	0.88 ± 0.07 [11]
11	Step size (nm)	u	u	10.0 ± 1.1 [11]	11.2 ± 1.0 [11]

a All chicken constructs are HMM. All human constructs are short S1 except those used in *V*_max_ and motility measurements as noted Errors are standard deviation and u is unknown. Table is separated into subsets relating measurements: (i) in the absence of actin and detecting Pi release or movement of the relay helix (rows 1–6), (ii) in the absence of actin and detecting ATP hydrolysis (rows 7–8), and (iii) in the presence of actin and detecting access of nucleotide to the active site (rows 9–11).

b 0.2 *μ*M HMM, 20 mM KCl, 5 mM Mg^2+^, 1 mM EGTA, 1 mM ATP, 20 mM Mops (pH 7.0), [actin] 0–80 or 0–150 *μ*M, 23–25°C.

c 20 *μ*g/mL HMM, 8 mM KCl, 1mM MgCl_2_, 1 mM EGTA, 2 mM ATP, 1 mM DTT, 10 mM imidazole (pH 7.0), [actin] 0–100 *μ*M, 37°C.

d Standard assay after Sellers et al. [23] for HMM in 60 mM KCl and at 30°C.

## Abbreviations

BME: *β*-Mercaptoethanol
ELC: Essential light chain of myosin
HMM: Heavy meromyosin
i7: 7 Amino acid insert QGPSFSF in smooth muscle myosin loop 1
MHC: Myosin heavy chain
MYH11: Smooth muscle MHC gene
NST: Nucleotide-sensitive tryptophan W506 adjacent to the relay helix
RLC: Regulatory light chain of myosin
S1: Myosin subfragment 1
SMA: Smooth muscle myosin subfragment 1 lacking i7
SMB: Smooth muscle myosin subfragment 1 containing i7
sSMA-W506: Single tryptophan version of short SMA with W506 and sequence numbering from human SMA
sSMB-W506: Single tryptophan version of short SMB with W506
